# Recalls of Cardiac Implants in the Last Decade: What Lessons Can We Learn?

**DOI:** 10.1371/journal.pone.0125987

**Published:** 2015-05-11

**Authors:** Shixuan Zhang, Christine Kriza, Sandra Schaller, Peter L. Kolominsky-Rabas, National Leading-Edge Cluster Medical Technologies ‘Medical Valley EMN’

**Affiliations:** 1 Interdisciplinary Centre for Health Technology Assessment (HTA) and Public Health (IZPH), University of Erlangen-Nürnberg, Erlangen, Bavaria, Germany; 2 National Leading-Edge Cluster Medical Technologies “Medical Valley EMN”, Erlangen, Bavaria, Germany; University of Akron, UNITED STATES

## Abstract

**Background:**

Due to an ageing population and demographic changes worldwide, a higher prevalence of heart disease is forecasted, which causes an even higher demand for cardiac implants in future. The increasing high incidence of clinical adverse events attributed especially to high-risk medical devices has led an advocated change from many stakeholders. This holds especially true for devices like cardiac implants, with their high-risk nature and high complication rates associated with considerable mortality, due to their frequent use in older populations with frequent co-morbidities. To ensure patients’ safety, the objective of this study is to analyze different cardiac implants recall reasons and different recall systems, based on an overview of the recalls of cardiac implant medical devices in the last decade. On the basis of the results from this structured analysis, this study provides recommendations on how to avoid such recalls from a manufacturer perspective, as well as how to timely react to an adverse event from a post-surveillance system perspective.

**Methods and Findings:**

A systematic search of cardiac implant recalls information has been performed in the PubMed, ScienceDirect and Scopus databases, as well as data sources in regulatory authorities from 193 UN Member States. Data has been extracted for the years 2004-2014 with the following criteria applied: cardiac implant medical device recalls and reasons for recall, associated harm or risk to patients. From the data sources described above, eleven regulatory authorities and 103 recall reports have been included in this study. The largest cardiac implant categories include ICDs 40.8%, pacemakers 14.5% and stents 14.5%. Regarding the recall reasons, the majority of reports were related to device battery problems (33.0%) and incorrect therapy delivery (31.1%). From a total of 103 recall reports, five reported death and serious injuries. Our review highlights weaknesses in the current cardiac implant recall system, including data reporting and management issues and provides recommendations for the improvement of safety information and management.

**Conclusion:**

Due to the mortality associated with the nature of cardiac implants, the traceability and transparency of safety hazards information is crucial. By a structured analysis of recall reasons and their efficient management, important knowledge is gained to inform an effective safety-reporting system for monitoring the safety of cardiac implanted patients, ideally by building up cardiac implant registries worldwide in the future.

## Introduction

### Rationale

With the rapid growth of the medical device industry in the last few years, the increasing high incidence of clinical adverse events attributed especially to high-risk medical devices has led to a vocalized need for change from many stakeholders [1]. A premarket approval process is key to prevent risk and harm stemming from faulty devices [2]. However, it is difficult to know how a device will perform over the long term in a real world setting [3]. It is well known that a number of serious events in the implant area happened in early 2012, which started a discussion of safety issues and monitoring medical devices. In case of PIP implants, about 300,000 women in 65 countries were affected by faulty devices. The second incident in the field of medical device discussed was “metal on metal” (MoM) hip implants, whereby people from all over the world may have been exposed to dangerously high level of toxic metals from defective hip implants [4].

The dangers are especially problematic for devices like cardiac implants, with their high-risk nature and high complication rates associated with considerable mortality, due to their frequent use in older populations with frequent co-morbidities[5]. An effective post-market surveillance system would reduce the risk and harm associated with these devices. Although medical devices have made an important contribution to improve patients’ quality of life, numerous weaknesses in their premarket evaluation and post-market surveillance system have been persistent [6]. Learning from previous recall experience can enable the improvement of the safety-reporting system and the entire post-market surveillance system.

### Background information

#### Major cardiac implants categories

This study analyzes different cardiac implants categories; the focus is made on the following four major cardiac implants categories:

- An ‘Implantable Cardioverter Defibrillator (ICD)’ is an implant placed under the collarbone. It can detect dangerous arrhythmias, and respond with an electrical signal to restore a normal rhythm [7].
- A pacemaker is also implanted under the collarbone. A lead is placed from the device to the inside of the ventricle wall to keep the heart beating regularly [8].
- Cardiac Resynchronization Therapy (CRT), which is also implanted under the collarbone, has three leads and can sense dangerous arrhythmia, and then deliver a pulse to correct the rhythm back to normal. CRT has either ICDs or pacemaker inside, so it is called either CRT-ICDs or CRT-Pacemaker [9].
- A coronary stent is a very small wire mesh tube used to prop open an artery during angioplasty [10].

#### Definition and classification of medical device recall

According to the U.S. Food and Drug Administration (FDA), a recall is an action taken to address a problem with a medical device that violates FDA law [11]. Recalls are classified by the FDA to indicate the relative degree of health hazard presented by the product being recalled [12]:

- Class I: will cause serious adverse health consequences or death;
- Class II: may cause temporary or medically reversible adverse health consequences or where the probability of serious adverse health consequences is remote;
- Class III: is not likely to cause adverse health consequences [12].

In addition, in comparison to device regulation centralized at the national level by the FDA in the U.S, the EU provides guidance but directives are interpreted by national Competent Authorities and private Notified Bodies [13]. EU member states use a different recall system to the US FDA system, namely the “Field Safety Corrective Actions”[14]. A “Field Safety Corrective Action” is an action taken by a manufacturer to reduce a risk of death or serious deterioration in the state of health associated with the use of a medical device that is already placed on the market. Such actions, whether associated with direct or indirect harm, should be reported and should be notified via a Field Safety Notice [14].

### Objectives

To ensure patients’ safety, the objective of this study is to analyze different cardiac implants recall reasons and different recall systems, based on an overview of the recalls of cardiac implant medical devices in the last decade. On the basis of the results from this structured analysis, this study provides recommendations on how to avoid such recalls from a manufacturer perspective, as well as how to timely react to an adverse event from a post-surveillance system perspective.

## Methods

### Key research questions

According to the objectives of this study, the following research questions are addressed:

- What was the recall ratio of different categories of cardiac implant medical devices?
- What was the ratio of different recall reasons among cardiac implant medical devices?
- From the analyzed recall reports, which reported death and serious injuries events?

### Information sources and search for cardiac implant recalls

This study applies two methodologies to search for potentially relevant data, one through literature research in academic journals, and the other by searching publications in regulatory authorities’ data sources. A systematic search was performed for English articles with the following search terms: recall, cardiovascular, cardiac and medical device in the PubMed (Medline), ScienceDirect (EMBASE) and Scopus databases. Furthermore, data on cardiac implants recalls in the last decade were collected from data sources provided by different regulatory authorities from 193 United Nations Member States [15]. Google was used to identify the relevant regulation authorities first. The criteria for selecting recall data sources were identified by the author team:

- Having a safety-reporting system with medical device recall information;
- Providing each recall report with sufficient information, such time, recall classification, recall reason etc.;
- Limiting language to English, Chinese, and German.

In order to collect data of cardiac implant recalls in the last decade, from each selected data sources, recall reports have been extracted with the following criteria applied. The search results for English and German were identified from the viewed records title and recall reasons were independently reviewed and screened by two researchers. Based on the research questions identified by the author team, the data was analyzed through a descriptive and comparative data analysis. The criteria were made focusing on recall reason related to technical problems. We have excluded recall reports based on human errors, for example connected to the mix up of device series numbers.

- Time duration from 2004 to2014;
- The recall product was attributed to a cardiac implant medical device;
- Recall reasons were due to causing harms or risk to patients or having the potential to cause such risk and harm.

## Results

### Bibliographic research results

**Fig 1:**
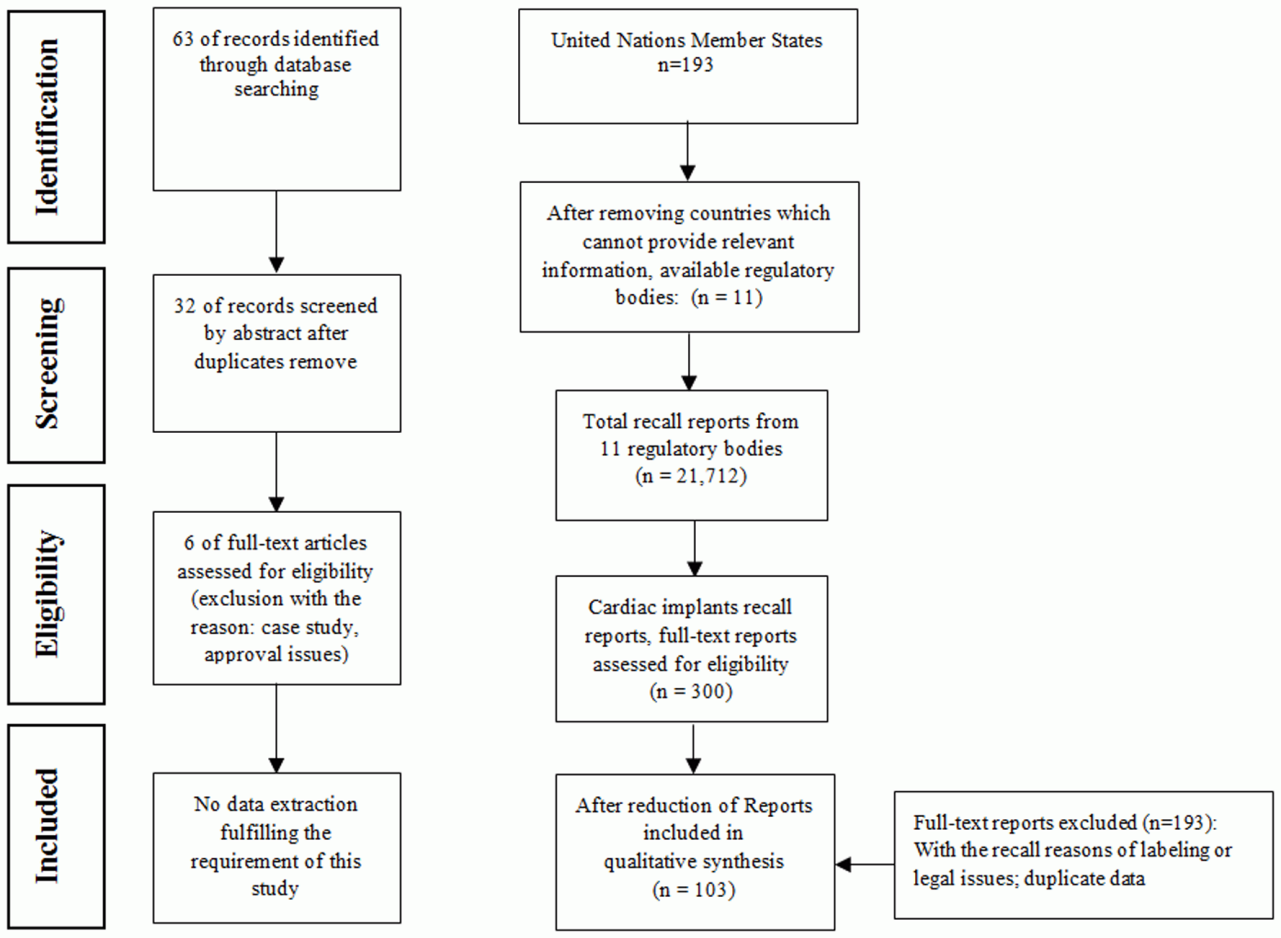
Flow Diagram. Fig 1 shows the process of selecting recall reports as Flow Diagram in line with the PRISMA guidelines. The Flow Diagram includes two search methodologies; the link one is the process of a systematic search performed for English articles in academic databases; the right one is from data sources provided by different regulatory authorities from 193 United Nations Member States. A total of 103 recall reports have been analyzed in this study.

The total number of recall reports identified from data sources in eleven regulatory authorities was 21,712. Of these, 300 recall reports were related to cardiac implants. After extracting the data based on the set criteria, and considering duplicate data, a total of 103 recall reports have been analyzed in this study. Six articles from scientific databases have been included for the full-text analysis, but none were included in the review as the data in these studies did not meet our described inclusion criteria. The process of selecting recall reports is shown as Flow Diagram in line with the PRISMA guidelines in Fig 1 [16]. Data sources from eleven regulatory authorities have been included in this study, which can be found in Table 1 as well as the full name of regulatory authorities.

**Table 1:** Number of cardiac implants and total recall reports.

Regulatory Authorities	Total cardiac implant recalls	Total recall report	Time period availability
U.S. Food and Drug Administration (FDA)	12	335	2004–2014
Canada. Health Canada (HC-SC)	10	2486	2005–2014
Australia. Therapeutic Goods Administration (TGA)	12	1050	2012–2014
New Zealand. Medicines and Medical Devices Safety Authority (Medsafe)	3	723	2012–2014
UK. Medicines and Healthcare Products Regulatory Agency (MHRA)	24	554	2004–2014
Ireland. Health Products Regulatory Authority (HPRA)	3	149	2004–2014
Switzerland. Swiss Agency for Therapeutic Products (Swissmedic)	67	3697	2005–2014
Germany. Federal Institute for Drugs AND Medical Devices (BfArM)	96	6632	2005–2014
PR China. China Food and Drug Administration (CFDA)	6	195	2010–2014
China Hong Kong Health Department	29	788	2005–2014
Saudi Arabia. Saudi Food and Drug Authority (SFDA)	38	5103	2011–2014

Table 1 indicates the number of recall reports in eleven regulatory authorities within the fixed time period, including total numbers of recall reports and total numbers of cardiac implants recall reports. This table aims to give readers an overall impression the medical device recalls situation.

### Recall ratio of different categories of cardiac implant medical devices

**Fig 2:**
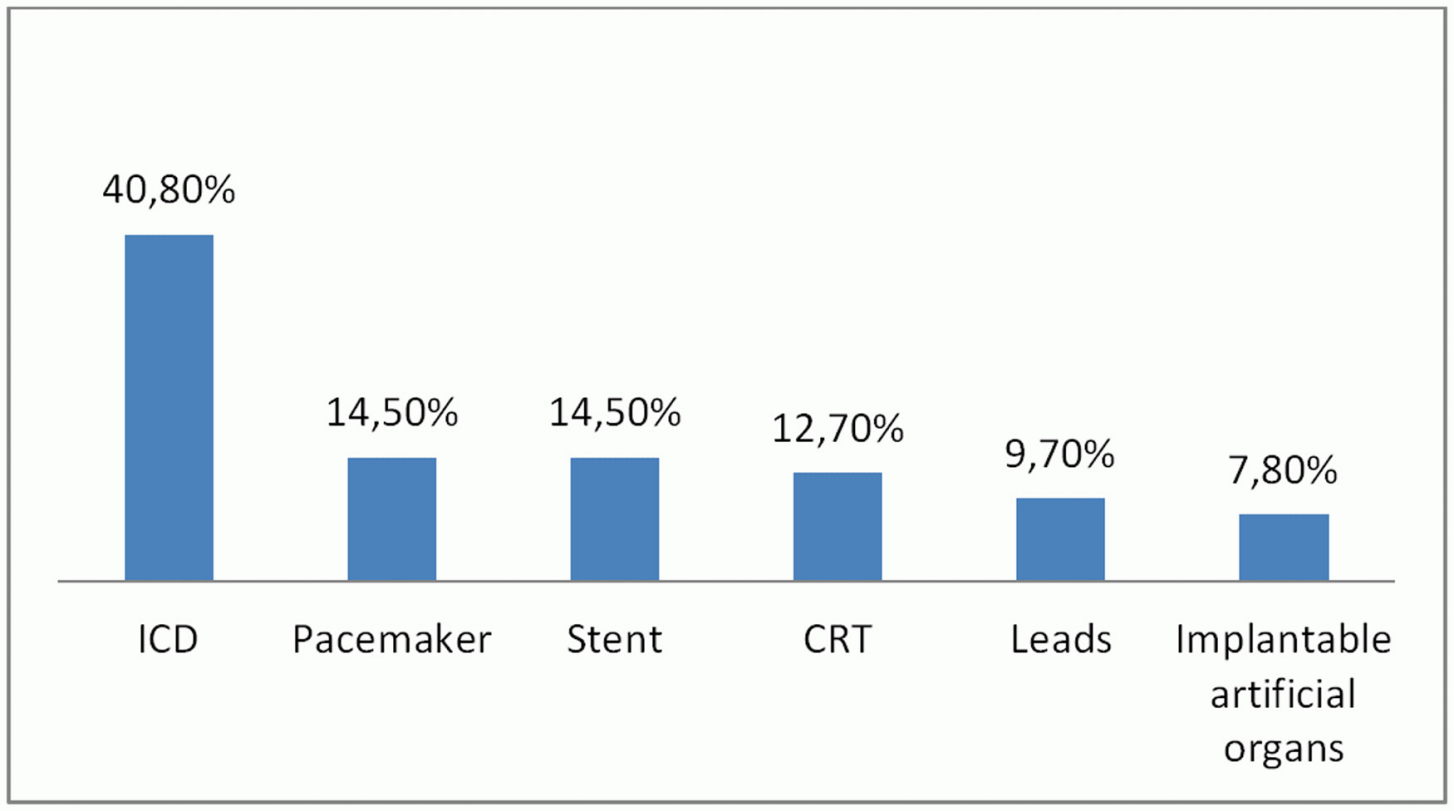
Product categories of recalled cardiac implants. Fig 2 indicates the recall rate of six different cardiac implant categories in the 103 recall reports analyzed in this study. Of all 103 recall reports, the ratio of categories is shown: ICDs 40.8%; pacemakers 14.5%; stents 14.5%; as well as CRTs 12.7%; leads 9.7% and implantable artificial organs 7.8%.

As shown in Fig 2, the 103 recall reports can be classified into six different categories: Implantable Cardioverter Defibrillators (ICDs), Cardiac Resynchronization Therapy (CRTs), pacemaker, coronary stent, leads and implantable artificial organs. Of all 103 recall reports, the ratio of categories is as follows: ICDs 40.8%; pacemakers 14.5%; stents 14.5%; as well as CRTs 12.7%; leads 9.7% and implantable artificial organs 7.8%.

### Recall reasons among cardiac implant medical devices

Regarding different problems causing the recall, 33.0% of the reports were related to problems with the device battery; 31.1% of devices were recalled due to incorrect therapy delivery; 15.5% devices had software problems; 14.6% devices had connection problems and 5.8% of devices did not deliver correct output data. The author group summarizes the problems of affected cardiac implant devices and formulates these into a classification system based on the guidelines of reporting devices problems from the FDA [17]. The recall reasons from 103 reports have been sorted into five categories and 27 sub-categories, which can be found in Table 2. More detailed information regarding the recall problems can be found in S1 Table. For each cardiac implant product category, recall reasons are illustrated in the following text, typical recall reports have been selected by the authors to highlight as examples, including detailed descriptions.

**Table 2:** Ratio of recall reasons among cardiac implant medical devices.

Categorizes	Sub-categorizes	ICD	CRT	Pacemaker	Stent	Leads	Implantable artificial organs	Total Number
Battery	Capacitor	10	6					16
	Voltage	2						2
	Connection	2	1				1	4
	Battery defect	3						3
	Reporting			2				2
	Premature battery depletion	4	2	1				7
Software	Performance inconsistency problem	5	1	1				7
	Inappropriately set	2	1	1				4
	Lead to battery defect	2		2				4
	Influence by environment			1				1
Output data	Incorrect express	1		1				2
	No output			1				1
	No or incorrect feedback						3	3
Therapy delivery	Background influence	1						1
	Pacing inhibition	4	1					5
	Inappropriate therapy	2		2				4
	Equipment malfunction	3	1		1			5
	Fractured				6	1		7
	Failed or partial deployment				6			6
	Leak			1				1
	Inadequate size				2		1	3
Connection	Weakened bond	1					1	2
	Partially or fully separated						1	1
	Separation of wires			2		2		4
	Bend relief						1	1
	Lead insulation abrasion					5		5
	Materials detached from guide wires					2		2
Total Number		42	13	15	15	10	8	103

Table 2 indicates that the recall reasons or problems of these 103 reports analyzed in this study. Based on the guidelines of reporting devices problems from the FDA, the author group summarizes the problems of affected cardiac implant devices and formulates these into a classification system. The recall reasons from 103 reports have been sorted into five categories and 27 sub-categories. The readers can find the specific problem description of each report in S1 Table.

For each cardiac implant product category, recall reasons are illustrated in the following text, typical recall reports have been selected by the authors to highlight as examples, including detailed descriptions.

#### Implantable cardioverter defibrillator (ICD)

Of all 42 recalled ICD reports, 50% had battery problems, 23.8% had problems related to therapy delivery, and 21.4% were associated with software malfunctions. One report was related to data output express issues, and another report was related to connection issues. Ten out of 21 reported battery problems recalls were due to capacitor issues. The latest recall was mentioned by Medicines and Healthcare Products Regulatory Agency (*MHRA*) in Sep. 2013, until recall issuing date, 38,500 ICDs and CRT-Ds have been distributed worldwide. The recall reason was due to the accelerated degradation of the capacitors, which in turn depleted the battery at a faster rate, thereby increasing the risk of faulty therapy delivery [18].

#### Pacemaker

Of all 15 reported pacemaker recalls, 33.3% involved a software malfunction, 20.0% had battery problems, and 20.0% were associated with therapy delivery inhibition. Two associated with delivering incorrect output data, another two with connection problems. Regarding the software malfunction issue, the latest recall was issued by Swiss Agency for Therapeutic Products (*Swissmedic*) in Dec. 2012. Approximately 8,200 of such devices have been distributed worldwide until the recall issuing date, and approximately 6,000 of these were estimated to be implanted. The related pacemakers did not provide a change in sensor driven (rate responsive) pacing rates in response to patient’s physical activity [19].

#### Coronary stent

15 recalled coronary stent reports were included in this study, whereas all of the recall reasons were associated with ‘therapy delivery problems’. Six reports were due to the fracture of the device; seven reports referred to a partially deployed or failed therapy, and two reports were associated with the inadequate size of the coronary stent. In July 2004, *FDA* issued two Class I recalls of coronary stents (TAXUS stent and Express^2^ stent). The recall involved approximately 85,000 TAXUS stent systems out of 500,000 in total which have been distributed worldwide until the recall issuing date. Approximately11, 000 Express^2^ stent systems were affected from a total of 600,000 distributed worldwide. The manufacturer received reports of one death and 18 serious injuries associated with the TAXUS stent systems, and two deaths and 25 serious injury reports related to the Express^2^ stent systems[20]. The recall reason was because of the characteristics in both design did not allow the balloon to deflate, which resulted in the impeded removal of the balloon after stent placement [21, 22].

#### Cardiac resynchronization therapy (CRT)

This review included 13 recall reports of CRTs, whereby nine reports were recalled due to battery issues, two reports were associated with software problems, and two reports with therapy delivery problems. Of all nine reported battery issue reports, six had capacitor malfunctions, and two experienced premature battery depletion. The latest recall was issued by Federal Institute for Drugs AND Medical Devices (*BfArM*) in Aug. 2013. A total of approximately 264,000 identified devices have been distributed and implanted, especially a subset of 38,500 devices that was manufactured prior to Dec. 2009 and has experienced a higher number of low voltage (LV) capacitor malfunctions. This referred to approximately 0.67% of the overall affected rate; the affected rate of other 225,500 devices was approximately 0.0093%. The reason for the recall was that the manufacturer identified a low voltage (LV) capacitor component in some devices, which may lead to diminished performance after two or more years from the time of implantation [23].

#### Leads

A total of ten recall reports associated with the malfunctioning of leads were included in this study. Nine had a recall reason related to connection problems, and one was due to an issue with the fracture of the device. Of the nine cases associated with connection problems, two were due to a separation of wires, five to a lead insulation abrasion, and two others were due to coating materials detaching from guide wires. The latest recall was issued by *Swissmedic* in Dec. 2012. The manufacturer confirmed 30 reports out of a total 13,500 devices implanted worldwide, where the analysis of the returned leads identified internal insulation breach under the right ventricular (RV) and the Superior Vena Cava (SVC) defibrillation coil electrode, resulting in low pacing impedance, ventricular oversensing and inappropriate therapies[24].

#### Implantable artificial organs

Our analysis identified a total of eight reports concerning implantable artificial organs. Three of them were related to inability to provide correct technical feedback data, four had connection problems, and one was due to the inadequate size of the device. The latest recall was issued by Therapeutic Goods Administration (*TGA*) in Jan. 2014 due to the high potential of implanting oversized valves. The rate of oversized valves occurred at a rate of 0.33%[25].

### Reporting adverse events causing deaths or serious injuries

Out of 103 analyzed recall reports, five reported death and serious injuries. According to the FDA’s definition, a ‘serious injury’ is an injury or illness that is life-threatening, results in permanent impairment of a body function, or permanent damage to a body structure, or necessitates medical or surgical intervention to preclude any of the above [26]. The five reports referred to the time period between2004 and 2011 and had implications in a number of countries worldwide. Table 3 provides the overview of the adverse events, reporting death and serious injuries identified in our analysis.

Four recalls reported death, with one being due to 'abrasion of the leads insulation’, which caused three deaths in 2011 [27]. Of these, one stent recall that was the result of the deflation failure that impeded the removal of the balloon after stent placement, which caused three deaths and 43 injuries in 2004.[21, 22] One recall was related to leads which was caused by a small number of fractures on the device being detected, causing five deaths in 2007 [28]. A further ICD recall stemmed from unexpected charge circuit time-outs or charge circuit inactive conditions in suspected cases, which caused four deaths in 2004 [29]. In addition, one stent case caused two serious injury events because of a detachment of the tip from the stent delivery system in 2008 [30].

**Table 3:** The death and serious injury reports from 103 identified recall reports.

Recall Date	Product Name	Announced Regulatory Authority	Distribution	Volume	Effect
2011/11/28	Riata and Riata ST Silicone Endocardial Defibrillation Leads	FDA, Swissmedic,BfArM	Worldwide	79,000 implanted worldwide	3 deaths and 2 serious injuries
2008/06/23	Stent, cardiovascular	Swissmedic,	Worldwide	7 complaint reports	2 serious injuries
2007/10/15	Sprint Fidelis Defibrillator Leads	FDA, HC-SC IMB, MHRA, Swissmedic, China HK, BfArM	Worldwide	268,000 implanted worldwide	5 deaths
2004/07/01	Express2paclitaxel drug-eluting & bare metal coronary stent system	FDA	Worldwide	Model 1: 500,000 manufactured, recall 85,000Model 2:600,000 manufactured, recall 11,000	Model 1: 1 death and 18 injured;Model 2: 2 deaths and 25 injuries
2004/04/04	Micro Jewell II Model 7223Cx and GEM DR Model 7271(ICD)	FDA, MHRA	Worldwide	6,268 manufactured, 1,800 implanted	1 serious injuries and 4 deaths

Table 3 provides the reports reporting death and serious injuries identified in our analysis, which aimed to highlight high-risk nature and high complication rates associated with considerable mortality of cardiac implants. Table 3 includes five recall reports, for each one, the readers can find ‘recall date’, ‘product name’, ‘announced regulatory authority’, as well as ‘distribution’, ‘volume’, and ‘Effect’.

## Discussion

This study summarizes cardiac implant recall reports worldwide in the last decade, identifies different recall reasons of each cardiac implants category, and highlights adverse events causing serious injuries and death. Through the search and data analysis process, the authors found that there several opportunities for improving the safety system for cardiac implants. One is from the manufacturers’ perspective, to avoid adverse events through summarizing the cardiac implants recall reasons. The other is from a regulatory authorities’ perspective, to timely react to an adverse event by overcoming weaknesses of the post-surveillance system. The authors provide recommendations to both manufacturers and authorities in the following texts.

### Recommendations for manufacturers to address focus areas associated with adverse events

Regarding different problems causing the recall, major problems were related to the device battery. Most cases had problems of premature battery depletion, some caused by the capacitor, some caused by the voltage. The device transferred to device end of life (EOL) without prior observation of elective replacement indication (ERI) even though battery capacity remains available. From most cases in the last decade, especially most cardiac implants such as ICDs, CRTs and pacemakers all have batteries inside; to prevent temporary or permanent loss of therapy caused by battery problems [18]. It is necessary for the manufacturers to check batteries after a defined time period.

Because of the characteristics of ICDs, CRTs and pacemakers, the problems related to the software and data output also need to be addressed by manufacturers. For stent design, it is necessary to prevent the fracture of the stent and the defect of the balloon [21, 22]. For implantable artificial organs, over-sizing of the materials was a common problem. Connection problems happened in most cases related to the lead, which connects the device to the inside of the ventricle wall. Especially the insulation of the leads should be addressed by the manufacturers, which can cause specific therapy delivery problems[27]. Further detailed information regarding these recall reasons and the number of recalled cardiac implants due to each reason can be found in Table 2 and S1 Table.

### Recommendation on how to improve the management of adverse events

#### Weaknesses found in the current cardiac implant recall system

To ensure patients’ safety in relation to cardiac implants, providing safety information to the public and relevant stakeholders such as patients, relatives, physicians, regulatory authorities, and HTA bodies is a basic requirement. The system for the provision of such safety information warrants further improvement from the current status quo. During the research process we identified that only eleven regulatory authorities provided detailed information regarding recalls in their medical device safety information (including regular updates), which were accessed in English, German or Chinese by the author group. Out of 39countries (34 OECD countries (The Organization for Economic Co-operation and Development)[31]and five BRICs countries (Brazil, Russia, India, China and South Africa)[32]), three regulatory authorities provided English data, additionally to reporting in the local language.

For the purpose of investigating different recall management styles among regulatory authorities, the author group chose one adverse event report, which was reported commonly in seven different regulatory authorities. The chosen recall report refers to the Sprint Fidelis Defibrillator Leads manufactured by *Medtronic*, which was recalled in Oct. 2007. Table 4 summarizes recall information in details from seven different national regulatory authorities [28, 33–38].

**Table 4:** Different recall management according to the same recall event in different regulatory authorities.

Regulatory Authority	Recall Date	Recall Level	Volume	Effect	Recall Reasons	Background Information	Patient contact Methods	Outcome of the recall
U.S. FDA	2007/10/15	Class I	268,000 implanted worldwide, 172,000 in U.S	less than 1% defected, no specific number of deaths and injuries	yes	yes	Toll-free number	no
Canada. HC-SC	2007/11/05	Recall	268,000	5 deaths, none in Canada	yes	yes	Phone	no
Ireland. IMB	2007/10/15	Recall	1,178 implanted in Ireland	N/a	yes	yes	phone	no
UK. MHRA	2007/10/19	Immediate action	6900 leads distributed in the UK	23 reports of leads fracture in UK	yes	yes	phone	Deadline (action complete): 28.12.2007
SwitzerlandSwissmedic	2007/10/19	Recall	268,000 implanted worldwide	665 chronic fractures in returned leads	yes	yes	Phone	no
China HK	2007/10/15	Recall	More than 200 distributed in HK	no serious injury or death in HK	yes	no	phone	no
Germany BfArM	2007/10/15	Field Safety Corrective Action	268,000implanted worldwide, 16,000 in Germany	665 chronic fractures in returned leads, 350 fractured in Germany	yes	yes	phone	no

Table 4 provides a comparison on different recall management strategies according to the same recall event in different regulatory authorities. And the chosen recall is the Sprint Fidelis Defibrillator Leads manufactured by *Medtronic*, which was recalled in Oct. 2007. The comparison contents includes ‘recall date’, ‘recall level’, ‘volume’, ‘effect’, as well as ‘recall reasons’, ‘background information’, ‘patient contact methods’ and ‘outcome of the recall’.

From a comparison of provided information, the following issues can be identified:

- Recall level: all regulatory authorities defined this recall at the highest level of the recall classification.
- Volume and effect: *FDA* and *BfArM* provided more detailed information with the number distributed worldwide in total and the local country, also regarding the ratio of defective devices. Other regulatory authorities provided the number distributed in local countries only.
- Only Health Canada (*HC-SC*) has published that there were five death reports involved in connection to this event.
- All regulatory authorities explained recall reasons and risks related to the affected device.
- Except China Hong Kong Health Department (*China HK*), all regulatory authorities provided background information on the affected device for stakeholders.
- Contact persons and telephone numbers have been provided for affected stakeholder groups by every regulatory authority.
- Outcome of the event treatment: only *MHRA* identified the exact date by which the recall had been completed.

#### Recommendations for the improvement of the safety information provision and management

In accordance with the results from a study by Kramer et al., referring to limits regarding lack of data on safety outcomes in both US and EU settings, we would like to propose some essential components to be included in a recall report [39]. First of all regarding a unique report name, it is recommended to take the name of recall report from the manufacturing company. This will enable reporting from each regulatory authority according to a unique report name. Secondly, recall reports should highlight the affected product category. Third, the recall issuing date and the deadline for completing withdrawals should be a necessary component of a recall report. This is especially important in the context of indicating the time period of completing the recall, which is missing in most current recall reports. The fourth is that recall classifications should be provided to highlight the risk level of affected devices. It is recommended that each regulatory authority provides a clear description and explanation of their recall classification system. Fifth, providing clear recall reasons and background information can help stakeholders to get a better understanding of the recall situation. Providing information on risk factors is to avoid adverse events occurring in the future in the context of patient safety. The sixth recommendations relates to the volume of affected devices distributed and implanted worldwide and in local countries, for the sake of comprehensive reporting. Seventh, death and injury reports should also be highlighted in the recall reports as a crucial aspect of protecting and ensuring patient safety. The last recommendation refers to the outcome of the recall management including the final effect and completion date, which should be added to recall reporting.

In the study by Kramer et al., it has been reported that the structure of the reporting system in most countries like US, EU, China and Japan is based on recall date of cardiac implants [39]. The use of a centralized reporting system is the current base of the recall system. We propose one additional feature to this useful concept in order to further strengthen reporting. For an improved overview and understanding of recall reasons, an additional product-based recall classification system as shown in the structured analysis is recommended by our author team. The results of this study can be taken as a small scale example of a safety-reporting system. The 103 cardiac implant recall reports analyzed in this study can be sorted into six product categories shown in Fig 2. The cardiac implant recall reasons can be sorted into five categories and 27 sub-categories. When inputting data into the database, it is recommended that under each product category, the recall reasons (plus sub-categories) are highlighted for each recalled medical device, as indicated in Table 2. Based on the product-category to improve a safety-reporting system, the provision of clear information would benefit patients, physicians and researchers and other stakeholder groups in the context of patient safety. For patients, information on recalls is comprehensive and accessible. Such a system could help physicians and patients to make more informed decisions when deciding about diagnosis and treatment measures. For the manufactures and researchers it aids in the analysis and comparison of the recall reasons of each product category, as well as the recall reason ratio.

As highlighted by Silva et al. and his colleague, there is an increasing importance of medical device registry especially registry for pacemaker, meanwhile registries provide re-usable data for researchers and relevant stakeholders [40]. It is recommended to implement implant registries in the context of a post market surveillance system. For example, as of September 2012, there were 38 cardiovascular device registries in the EU [41]. An implant registry is a tool to deliver long-term observational data related to the performance of medical devices, but can also be regarded as an overriding tool when it comes to controlling the high incidence of adverse events[41]. Although registries do not handle recall management per se, they have played an important role in the quick identification of patients, when a recall or adverse event occurred [42]. In addition, registries can survey large populations, providing a powerful scientific method for long-term observational data collection [43]. The authors’ recommendations can provide helpful tools for the use of implant registries in the context of a post market surveillance system. In addition, implant registries can facilitate communication among different stakeholders. For example, within the EU, the European Heart Rhythm Association (EHRA) takes a role in helping to coordinate dialogue among stakeholders. Some unexpected performance of medical devices has been identified early to improve patient safety [44]. A recent development includes plans for a compulsory-nation-wide registry for all medical implants in the Netherlands, based on legal provisions established by the Dutch parliament [45].

In addition to the important issue of patient safety, the consideration of the economic impact of recalls is important. As such, economic losses on the industry and health care payer side can be significant when large numbers of medical devices are recalled. Further, the negative impact resulting from a recall on the reduction of industry market shares needs to be considered. It is estimated that *Medtronic*’s Fidelis recall cost Medicare (The Official U.S. Government Site for Medicare) some $287 million over five years for monitoring or replacing the leads[46]. Another example that the case of *Johnson & Johnson* suffering significant losses with a decrease of 7.7% in revenue over the previous year in 2010 because the company had recalled various products due to problems with their quality [47]. Therefore, it is important for manufacturers, insurance companies and the health care system overall to invest in a strong monitoring and surveillance system of medical devices in order to make industry investments more efficient and less risky, in addition to protecting patient safety. Registries play an important role in a strong surveillance system.

### Study limitations

There are several limitations in this study. First of all, the authors were only able to review data in the English, German and Chinese language. As a result, there could potentially be important data missing from other countries that do not provide information in these languages. In addition, because of lack of language capacities, the report selection and data extraction from each recall report could not be done by two researchers independently, i.e. Chinese recall reports could only be analyzed by one researcher and translated to the other group members.

## Conclusions

Due to the mortality associated with the nature of cardiac implants, the traceability and transparency of safety hazards information is crucial. Through a structured analysis, important knowledge is gained that can inform an efficient safety-reporting system for monitoring the patients’ safety. Due to an ageing population and demographic changes worldwide, a higher prevalence of heart disease is forecasted, which causes an even higher demand for cardiac implants in future. The public and relevant stakeholders such as physicians, manufacturers, regulatory authorities and HTA bodies should be prepared to appropriately deal with related issues in the context of patient safety.

## Supporting Information

S1 Table Detailed information of recall problems description of each recall report. S1 Table is an extension of Table 2, which includes the detail information such as recall problems of each recall report identified in this study.
(DOCX)
