# Supplementary material for: Recalls of Cardiac Implants in the Last Decade: What Lessons Can We Learn?
Source: PLoS One. 2015 May 11;10(5):e0125987. doi: 10.1371/journal.pone.0125987 (PMC4427435; doi:10.1371/journal.pone.0125987)
Supplement: S1 Table — is an extension of Table 2, which includes the detail information such as recall problems of each recall report identified in this study. (DOCX) [file pone.0125987.s001.docx]

| **Supplementary Table 1. Recall reasons analysis of cardiac implants** | | | | | | | | |
| --- | --- | --- | --- | --- | --- | --- | --- | --- |
| categorizes | sub-categorizes | Recall reason in details | ICD | CRT | Pacemaker | Stent | Leads | Implantable artificial organ |
| Battery | Capacitor | Some of the devices with suspect capacitors have had unexpected charge circuit time-outs or charge circuit inactive conditions as the battery voltage nears the level for Elective Replacement of the devices.[[1](#_ENREF_1)] | 10 |  |  |  |  |  |
|  |  | A rare condition in which an internal protective fuse can be unintentionally activated while the device is charging its capacitors for shock delivery or induction. Should this occur, defibrillator would not be able to deliver therapy. [[2](#_ENREF_2)] |  |  |  |  |  |  |
|  |  | A rare condition in which an internal protective fuse can be unintentionally activated while the device is charging its capacitors for shock delivery or induction. The defibrillator may not be able to deliver therapy or communicate with the programmer, and may be unable to emit tones or otherwise respond to magnet application. [[3](#_ENREF_3)] |  |  |  |  |  |  |
|  |  | Specific low-voltage capacitors from a former supplier may be subject to degradation, which may cause accelerated battery depletion and may reduce the time between elective replacement indicator (ERI) and battery end of life (EOL) to less than three months. [[4](#_ENREF_4)] |  |  |  |  |  |  |
|  |  | Potential for reduced ERI to EOL time due to low-voltage capacitor degradation in a subset of ICDs and CRT-Ds.[[5](#_ENREF_5)] |  |  |  |  |  |  |
|  |  | Risk of loss of CRT/ICD therapy due to rapid battery depletion as a result of capacitor degradation occurring in a subset of devices. [[6](#_ENREF_6)] |  | 6 |  |  |  |  |
|  |  | Five reports regarding capacitors have had temporary or permanent degradation, premature battery depletion occur.[[7](#_ENREF_7)] |  |  |  |  |  |  |
|  |  | Premature battery depletion may occur because of capacitor damage in a series CRT-Ds and ICDs. [[8](#_ENREF_8)] |  |  |  |  |  |  |
|  |  | The devices may not meet expected device longevity due to gradually increasing current drain caused by low voltage capacitor degradation. This issue may present in the affected devices as reaching the Recommended Replacement Time (RRT) earlier than projected. [[9](#_ENREF_9)] |  |  |  |  |  |  |
|  |  | Boston Scientific has determined that the performance of a low voltage capacitor in this subset of devices may be compromised over time, causing increased current drain that can lead to premature battery depletion. [[10](#_ENREF_10)] |  |  |  |  |  |  |
|  |  | Some capacitors from specific lots may perform in a manner that leads to device malfunction, including intermittent or permanent loss of therapy or premature battery depletion. [[11](#_ENREF_11)] |  |  |  |  |  |  |
|  | Voltage | Technical investigations confirmed that the observed drop in the battery voltage is available with an unexpected, persistent minimal current flow in context, which can only occur in transport mode, in which the aggregates are up to the time of implantation.[[12](#_ENREF_12)] | 2 |  |  |  |  |  |
|  |  | Premature battery depletion and/or prolonged charge time may occur in a limited number of alto implantable cardioverter defibrillator.[[13](#_ENREF_13)] |  |  |  |  |  |  |
|  | Connection | Deterioration in a wire insulator could cause a short circuit, resulting in the devices' inability to deliver an electrical shock during episodes of arrhythmia. [[14](#_ENREF_14)] | 2 | 1 |  |  |  |  |
|  |  | The ICDs cannot deliver shock for therapy properly because the circuit board connection temporary or permanent unstable. [[15](#_ENREF_15)] |  |  |  |  |  |  |
|  |  | We have noticed that may arise in the course of use by an initial damage to the cable sheath during implantation cause damage to the cable. [[16](#_ENREF_16)] |  |  |  |  |  | 1 |
|  | Battery defect | Potential battery defect; possible rapid depletion, prevalence unknown. [[17](#_ENREF_17)] | 3 |  |  |  |  |  |
|  |  | The affected batteries may have a shorted battery cell, which would lead to a reduction of the total capacity of the battery. [[18](#_ENREF_18)] |  |  |  |  |  |  |
|  |  | These defibrillators include a particular electrical component may fail. Such a failure could cause a short circuit through which would prematurely discharge the battery in the defibrillator. A defibrillator is no longer functional when the battery is fully discharged. [[19](#_ENREF_19)] |  |  |  |  |  |  |
|  | Reporting | This report indicated that the battery of the device was still in the "Begin Of Life (BOL) function start" condition and an adequate residual-operating system runtime (e.g. several years) was shown. In case this inconsistency resulted in unnecessary explanation of the device. [[20](#_ENREF_20)] |  |  | 2 |  |  |  |
|  |  | As is the possibility of a faulty display and alarm reporting a low electrode impedance value. [[21](#_ENREF_21)] |  |  |  |  |  |  |
|  | Premature battery depletion | They may not operate for as long as expected; They may not operate for the full timeframe of at least three months between the alert for it to be replaced [the Elective Replacement Indicator (ERI)] and the End of Life (EOL) alert.[[22](#_ENREF_22)] | 4 |  |  |  |  |  |
|  |  | Risk of S-ICD being unable to deliver shock for therapy after elective replacement indicator (ERI) audible warning. The ERI may occur earlier than expected and the end of life (EOL) indicator may occur before the nominal 3 months.[[23](#_ENREF_23)] |  |  |  |  |  |  |
|  |  | Patients with relevant pacemakers or CRT-Ps may experience a temporary or permanent loss of therapy, telemetry, or premature battery depletion. Patients with ICDs in question can learn an incorrect detection or premature battery depletion. [[24](#_ENREF_24)] |  | 2 |  |  |  |  |
|  |  | Delay in delivery of therapy during device middle-of-life phase due to temporarily extended charge time limits; Transition to device end of life (EOL) without prior observation of elective replacement indication (ERI) even though battery capacity remains available. [[25](#_ENREF_25)] |  |  |  |  |  |  |
|  |  | Risk of loss of pacing due to overestimation of the time remaining to elective replacement indicator (ERI). [[26](#_ENREF_26)] |  |  | 1 |  |  |  |
| Software | Performance inconsistency | The manufacturer is informing you of an issue related to installation or removal of the Lead Integrity Alert (LIA) software in En Trust defibrillators. In those devices only, installation or removal of LIA will inadvertently turn off two audible patient alerts described below. This does not affect LIA or other device functionality. [[27](#_ENREF_27)] | 5 |  |  |  |  |  |
|  |  | The manufacturer has detected a risk of high voltage (HV) therapy not being available after anti-tachycardia pacing (ATP) in the ventricular tachycardia (VT) zone, as a result of a programmer software error that can lead to an unanticipated parameter change. High Voltage Therapy remains available in the VF detection zone. [[28](#_ENREF_28)] |  |  |  |  |  |  |
|  |  | Careful examination showed that this software can only occur anomaly at a rarely occurring sequence of events: The criterion for charging the capacitors shock (ventricular arrhythmias) and the criterion for the mode switch (atrial arrhythmia) are satisfied at exactly the same time. The unit is (because of sustained ventricular arrhythmia) delivers a shock. [[29](#_ENREF_29)] |  |  |  |  |  |  |
|  |  | The manufacturer is informing you of a rare device software issue in the ICD and CRT-D device models listed above. A software update will be available to correct this issue. The root cause to be a rare and specific sequence of events that must occur within a few milliseconds of each other: High voltage capacitors reach programmed energy (charge end); Battery voltage measurement in-process at charge end VT/VF rhythm self terminates and therapy is aborted.[[30](#_ENREF_30)] |  |  |  |  |  |  |
|  |  | The manufacturer has found that under certain conditions the biphasic with the above software versions can only give 100 joules to a patient with defibrillation, which is lower than the minimum recommended factory default setting of 200 J. [[31](#_ENREF_31)] |  |  |  |  |  |  |
|  |  | This letter is to inform you about a software anomaly in Vitatron dual-chamber pacemakers, the C-and T-series. If these devices are programmed for certain parameters, the software anomaly may manifest clinically in a reset of the pacemaker or in the absence of stimulation, ie when the underlying rhythm of the patient falls below the programmed lower rate. [[32](#_ENREF_32)] |  |  | 1 |  |  |  |
|  |  | A rare indicator of the software has been shown while the dual-chamber cardiac pacing at the same time. [[33](#_ENREF_33)] |  | 1 |  |  |  |  |
|  | Inappropriately set | When using the Merlin Programmer with software version17.2.2 as part of a single VF detection zone configuration for ICD/CRT-D devices, the sinus redetection value will be inappropriately set to zero milliseconds. As a result, any intrinsic activity following the first shock will be considered a “sinus rate” and the device will diagnose “return to sinus”. [[34](#_ENREF_34)] | 2 | 1 |  |  |  |  |
|  |  | Measurement error will lead to set “zero” for the battery voltage, and achieve Elective Replacement Indicator. [[35](#_ENREF_35)] |  |  |  |  |  |  |
|  |  | The manufacturer has identified that a subset of Accent SR single chamber model PM1110 and Accent DR dual chamber model PM2112 pacemakers will not provide a change in sensor driven (rate responsive) pacing rates in response to patient physical activity due to an incorrect software setting. [[36](#_ENREF_36)] |  |  | 1 |  |  |  |
|  | Lead to battery defect | The manufacturer sets all customers of the fact that the software version 2.002 and earlier versions of AEDs, the DDU-series, under certain circumstances, the warning "Low Battery" and "Replace Battery" does not indicate the previously determined by an automatic self-test. In this case, the user may not be aware of the almost dead battery and the device is not able to release a shock. [[37](#_ENREF_37)] | 2 |  |  |  |  |  |
|  |  | The wrong indicators of voltage and Elective Replacement Indicator have been reported because of the software issues. [[38](#_ENREF_38)] |  |  |  |  |  |  |
|  |  | Following a software update the new battery impedance elective replacement indicator (ERI) threshold may trigger an unexpected ERI in some EnRhythm® and EnRhythm MRI™ devices. [[39](#_ENREF_39)] |  |  | 2 |  |  |  |
|  |  | A programmer software anomaly can lead to incorrect reporting of battery voltage, expected battery longevity and Elective Replacement Indicator (ERI) status in certain St. Jude Medical pacemakers. The anomaly does not affect the device’s actual battery voltage, longevity or functionality, but could result in inaccurate reporting of the status of these measured data parameters. [[40](#_ENREF_40)] |  |  |  |  |  |  |
|  | Influence by environment | The manufacturer has identified that a small subset of 2090 Programmers have an incorrect software configuration for the country in which that are physically located. When a programmer is manufactured, it is configured for the region in which it will be located. [[41](#_ENREF_41)] |  |  | 1 |  |  |  |
| Output data | Incorrect express | Device delivers the proper charge, but prints the wrong number on the programmer. [[42](#_ENREF_42)] | 1 |  |  |  |  |  |
|  |  | The device may display an “electrical neutral line” which could be misinterpreted as an asystoly of the patient. Reboot of the Monitoring Unit if the view number 4 is selected and the realtime printout is being started. [[43](#_ENREF_43)] |  |  | 1 |  |  |  |
|  | No output | The manufacturer observed that a no output condition could occur in a limited number of Symphony or Rhapsody pacemakers. The no-output condition could occur due to metal migration caused by a specific manufacturing process.[[44](#_ENREF_44)] |  |  | 1 |  |  |  |
|  | No or incorrect alarm | An investigation of a single customer report has confirmed that it is possible in a specific circumstance for medium priority (Low Flow, High Power, and Suction) alarm limits to be exceeded without an audible notification or a visible alarm message being displayed. [[45](#_ENREF_45)] |  |  |  |  |  | 3 |
|  |  | A recent incident involving a patient death thirty-four months post. Log file analysis confirmed that the pump had stopped. Functional testing of the returned controller showed that the device (including motor control circuits) performed all functions as intended within specification and with no fault alarms or errors. The exact cause of the event cannot be conclusively determined. [[46](#_ENREF_46)] |  |  |  |  |  |  |
|  |  | It is possible in a specific circumstance for medium priority alarm limits to be exceeded without an audible notification or a visible alarm message being displayed. [[47](#_ENREF_47)] |  |  |  |  |  |  |
| Therapy delivery | Background influence | A particular vendor-supplied memory chip can be affected at a low frequency rate by background levels of atmospheric ionizing cosmic radiation ("background cosmic radiation"). The anomaly can trigger a temporary loss of pacing function and permanent loss of defibrillation support. [[48](#_ENREF_48)] | 1 |  |  |  |  |  |
|  | Pacing inhibition | Potential for reduction in number of shocks (skipped charge) delivered per therapy episode. Potential for inappropriate rate responsive pacing for up to 90 minutes during automatic and clinician initiated capacitor reformation. [[49](#_ENREF_49)] | 4 |  |  |  |  |  |
|  |  | Risk of inappropriate shocking, pacing inhibition or shocking inhibition due to internal insulation abrasion. [[50](#_ENREF_50)] |  |  |  |  |  |  |
|  |  | The above-referenced CRT-Ds are programmed to LV-only pacing, it is possible for the patient’s intrinsic signals to cause the RV refractory period to be prematurely terminated. The premature termination of the RV refractory period can result in inappropriate sensing of intrinsic RV activity that would normally fall into refractory. [[51](#_ENREF_51)] |  | 1 |  |  |  |  |
|  |  | Risk of bradycardia / syncope due to pacing inhibition. Potential for pacing inhibition associated with ventricular oversensing if the follow-up monitoring feature, PhD, is programmed ON and the device is connected to high polarization defibrillation leads. [[52](#_ENREF_52)] |  |  |  |  |  |  |
|  | Inappropriate therapy | A printed circuit board assembly (PCBA) may cause an intermittent short. If this occurs, it may result in no therapy delivery or delivery of an incorrect defibrillation waveform. [[53](#_ENREF_53)] |  |  | 2 |  |  |  |
|  |  | There is a possibility that these make the decision to deliver a shock or no shock faulty if the semi-automated external defibrillator is set to auto analysis. [[54](#_ENREF_54)] |  |  |  |  |  |  |
|  |  | The manufacturer has recently determined that if the Respiratory Sensor is programmed On, such RV lead complications may cause additional oversensing, thereby increasing the probability of inappropriate therapy. Five to eight successive inappropriate shocks could leave the device unable to treat an actual arrhythmia until the current episode ends. [[55](#_ENREF_55)] | 2 |  |  |  |  |  |
|  |  | The manufacturer recently received four reports from Germany concerning the OPTIMIZER III IPG. One of the device components, the reed switch, became stuck during IPG recharge. When it became stuck the devices went into "magnet mode" which is a setting which automatically turns off Cardiac Contractility Modulation therapy. This state can typically be changed to reinitiate therapy by reprogramming the OPTIMIZER® III device with the OMNI Programmer. However, a stuck reed switch affects the telemetry reception by the IPG, preventing reprogramming until the reed switch is released. [[56](#_ENREF_56)] |  |  |  |  |  |  |
|  | Equipment malfunction | With this letter is to provide you with important safety information about a particular, but rarely applied implantation technique. Guidant reported two cases of equipment malfunction in connection with a rarely applied subpectoral implantation technique. The vast majority of these aggregates was implanted subcutaneously at the estimates and is therefore not affected by this disorder. [[57](#_ENREF_57)] | 3 |  |  |  |  |  |
|  |  | A recurrent mechanical stress on the titanium case lead to damage to components and thus to an equipment malfunction. [[58](#_ENREF_58)] |  | 1 |  |  |  |  |
|  |  | Recall due to component failure resulting in potential loss of cardioversion, defibrillation, and reduced device longevity. [[59](#_ENREF_59)] |  |  |  |  |  |  |
|  |  | To date, the manufacturer has received complaints regarding the inability to flush the device when purging the instrument. [[60](#_ENREF_60)] |  |  |  | 1 |  |  |
|  | Fractured | These leads are being recalled because a small number of fractures have been detected. When the lead breaks (fractures), it may cause inappropriate shocks or result in a loss of therapy, such as pacing or shocking. [[61](#_ENREF_61)] |  |  |  |  | 1 |  |
|  |  | A consistent increase in the internal scrap rate (failure mode: broken stent struts) of nitinol stents was observed. [[62](#_ENREF_62)] |  |  |  | 6 |  |  |
|  |  | One case is a partially deployed stent fractured upon attempting to retrieve the device from the patient, and a small surgical cut down procedure was performed to retrieve the fractured stent segment. [[63](#_ENREF_63)] |  |  |  |  |  |  |
|  |  | In the course of internal controls we found out that during the sterilization, a damage of the distal spring of the mounted implant might be happen. This damage could lead to a fracture and a reduced radial force in the distal spring of the stent graft. In this case the integrity of the stent graft would no longer be guaranteed. [[64](#_ENREF_64)] |  |  |  |  |  |  |
|  |  | A detachment of the tip from the stent delivery system has been reported, potential health hazard events resulting from this type of failure include increased procedure time, vessel wall injury, stoke and/or emergency surgery to remove the detached tip. [[65](#_ENREF_65)] |  |  |  |  |  |  |
|  |  | A potential issue with a specific subset where deployment complication may occur. This type of issue could potentially lead to a sub-optimal deployment resulting in serious deterioration to a patient’s health. [[66](#_ENREF_66)] |  |  |  |  |  |  |
|  |  | Two complaints have been reported in Europe for a detachment of the tip from the stent delivery system. Potential health hazard events resulting from this type of failure include increased procedure time, vessel wall injury and/or stent displacement during attempts to retrieve the tip. There is also a risk that the patient may require emergency surgery to remove the tip. [[67](#_ENREF_67)] |  |  |  |  |  |  |
|  | Failed or partially to deploy | During routine final product lot release testing, a NexStent Carotid Stent device failed to deploy when the outer catheter (proximal outer and distal sheath) did not pull back and expose the self-expanding stent. [[68](#_ENREF_68)] |  |  |  | 6 |  |  |
|  |  | Characteristics in the design of these two lots resulted in failure of the balloon to deflate and impeded removal of the balloon after stent placement. [[69](#_ENREF_69), [70](#_ENREF_70)] |  |  |  |  |  |  |
|  |  | The deployment mechanism for the affected LifeStent Solo Vascular Stents may not perform properly when used. Deployment issues range from failure to deploy, partial deployment, and difficult deployment. [[71](#_ENREF_71)] |  |  |  |  |  |  |
|  |  | The Innova™ Self-Expanding Stent System is indicated for the treatment of peripheral vascular lesions. The manufacturer has received 6 complaints involving no deployment or partial deployment of the Innova Stents. Potential health hazard events resulting from this type of failure include increased procedure time, vessel wall injury, and emergency surgery to remove the partially deployed stent. [[72](#_ENREF_72)] |  |  |  |  |  |  |
|  |  | It seems that a possible defect in the deployment of the balloon has led to some incident reporting for the above-mentioned codes. [[73](#_ENREF_73)] |  |  |  |  |  |  |
|  | Leak | A seal within the devices can leak, allowing moisture to affect the electronic circuits. [[74](#_ENREF_74)] |  |  | 1 |  |  |  |
|  | Inadequate size | The IntraStent is a peripheral / biliary stent. The manufacturer initiated the recall of certain lots of this product following the discovery that two symbols (the symbol for length and the symbol for diameter) on the side and end-flaps of IntraStent boxes in the affected lots are reversed. The length and diameter symbols on the top of the box and on the device pouch are correct. [[75](#_ENREF_75)] |  |  |  | 2 |  |  |
|  |  | Stent Expansion Uniformity refers to the percentage difference between the largest and smallest outer diameter measurement on a single stent deployed in an unconstrained manner to its rated burst pressure in engineering laboratory testing. [[76](#_ENREF_76)] |  |  |  |  |  |  |
|  |  | The Manufacturer has issued a hazard alert for its Mosaic Porcine Aortic Bioprosthesis Model 305 due to the potential for implanting oversized valves. [[77](#_ENREF_77)] |  |  |  |  |  | 1 |
| Connection | Weakened bond | The manufacturer has determined that the bond between the header and case could be weakened by significant forces associated with a subpectoral implant procedure or when a device in a subpectoral position is pushed against a rib during contraction of the pectoralis muscle. A weakened header bond may alter lead impedance and introduce noise that may inhibit pacing therapy or initiate inappropriate tachy therapy. Additional mechanical stress applied to a weakened bond may eventually cause header connection wires to fracture, resulting in loss of therapy. [[78](#_ENREF_78)] | 1 |  |  |  |  |  |
|  |  | The root cause of these events has been attributed to a weakened bond between the capsule containing the valve and the delivery shaft. There is no potential for a component to separate from the device (the valve capsule remains connected to the DCS inner lumen). However, the weakened bond could result in the inability to unsheathe and deploy the valve from the capsule. [[79](#_ENREF_79)] |  |  |  |  |  | 1 |
|  | Partially or fully separated | The HVAD Pump’s driveline connector housing became partially or fully separated from the front portion of the driveline connector. In the unlikely event of a separation, we advised that a repair is necessary. If left unattended, electrical connection to the controller could be affected and a VAD stop alarm could result. [[80](#_ENREF_80)] |  |  |  |  |  | 1 |
|  | Separation of wires | The Kappa and Sigma pacemakers in these identified series may fail due to a separation of wires that connect the electronic circuit to other pacemaker components, such as the battery. [[81](#_ENREF_81)] |  |  | 2 |  |  |  |
|  |  | May fail due to separation of interconnect wires from the hybrid circuit. This failure mechanism may present clinically as loss of rate response, premature battery depletion, intermittent or total loss of telemetry, or no output.[[82](#_ENREF_82)] |  |  |  |  |  |  |
|  |  | The manufacturer has identified that the device will lose function due to the separation of wire. When that happens, it may cause loss of therapy delivery, premature battery depletion, and temporary or permanent loss of signal. [[83](#_ENREF_83)] |  |  |  |  | 2 |  |
|  |  | The manufacturer has identified that the device will lose function due to the separation of wire. When that happens, it may cause loss of therapy delivery, premature battery depletion, and temporary or permanent loss of signal. [[84](#_ENREF_84)] |  |  |  |  |  |  |
|  | Bend relief | The manufacturer is aware of a recent trend in reports of disconnection of the bend relief from the sealed outflow graft, a component of the HeartMate II LVAS. The bend relief is a tube of ePTFE surrounding the outflow graft proximal to the pump that is designed to prevent kinking of the outflow graft. Disconnection of bend relief from the sealed outflow graft may potentially lead to outflow graft kinking and/or graft abrasion. Symptoms of outflow graft kinking included low pump flow, hemolysis, bleeding, and fluctuations in pump flow, speed and/or power, or worsening symptoms of heart failure. Graft abrasion may lead to serious bleeding. [[85](#_ENREF_85)] |  |  |  |  |  | 1 |
|  | Lead insulation abrasion | Failures associated with lead insulation abrasion on the St. Jude Medical Riata and Riata ST Silicone Endocardial Defibrillation Leads may cause the conductors to become externalized. [[86](#_ENREF_86)] |  |  |  |  | 5 |  |
|  |  | The manufacturer has confirmed that the analysis of the returned leads identified internal insulation breach under the right ventricular (RV) and Superior Vena Cava (SVC) defibrillation coil electrode, resulting in low pacing impedance, and/or ventricular oversensing and/or inappropriate therapies. [[87](#_ENREF_87)] |  |  |  |  |  |  |
|  |  | Risk of inappropriate shock or therapy failure due to wearing of lead insulation after implantation. [[88](#_ENREF_88)] |  |  |  |  |  |  |
|  |  | Risk of worsening heart failure symptoms due to wear and/or abrasion of lead insulation after implantation. [[89](#_ENREF_89)] |  |  |  |  |  |  |
|  |  | Lead abrasion failures identified in the Riata silicone insulated defibrillation leads as compared to our newer lead models utilizing the Optim® insulation material (Riata ST Optim and Durata® family of defibrillation leads). [[90](#_ENREF_90)] |  |  |  |  |  |  |
|  | Materials detached from guide wires | There is a potential for PTFE (polytetrafluroethylene) coating to delaminate and detach from guide wire. Medtronic steerable guide wires are used to aid in the placement of ventricular leads in the coronary vasculature. [[91](#_ENREF_91)] |  |  |  |  | 2 |  |
|  |  | We identified that the PTFE coating on the gold plated distal coil of the Back-up Meier Steerable Guidewires of the identified lots/batches have the potential for PTFE delamination. [[92](#_ENREF_92)] |  |  |  |  |  |  |

1. U.S. Food and Drug Administration. *Medtronic Announces a Nationwide, Voluntarily Recall of Small Subset of Two Implantable Cardioverter-Defibrillator Models*. 2004 19.Jun.2013; Available from: <http://www.fda.gov/Safety/Recalls/ArchiveRecalls/2004/ucm111586.htm>.

2. Government of Canada. *Subcutaneous Implantable Defibrillator*. 2013 30.May.2013 [cited 2013 01.Mar]; Available from: <http://www.healthycanadians.gc.ca/recall-alert-rappel-avis/hc-sc/2013/33707r-eng.php>.

3. Department of Health Hong Kong. *Medical Device Safety Alert: Boston Scientific subcutaneous implantable defibrillator system*. 2013 [cited 2013 12.Mar]; Available from: <http://www.mdco.gov.hk/english/safety/recalls/recalls_20130312.html>.

4. Swiss Agency for Therapeutic Products. *Expansion of the April 2007 Product Advisory regarding the potential for reduced ERI to EOL time due to low-voltage capacitor degradation*. 2009 [cited 2009 23.Mar]; Available from: https://[www.swissmedic.ch/recalllists_dl/00760/Vk_20090312_05-e1.pdf](http://www.swissmedic.ch/recalllists_dl/00760/Vk_20090312_05-e1.pdf).

5. Government of Canada. *For Health Professionals-Urgent Medical Device Information-Subset of Implantable Cardiac Defibrillators and Cardiac Resynchronization Therapy Defibrillators* 2007 05.Mar.2013 [cited 2007 05.Apr]; Available from: <http://www.healthycanadians.gc.ca/recall-alert-rappel-avis/hc-sc/2007/14457a-eng.php>.

6. UK. Medicines and Healthcare Products Regulatory Agency (MHRA). *Medical Device Alert: Implantable cardioverter defibrillators (ICD) and cardiac resynchronisation therapy devices (CRT-D) manufactured by Boston Scientific (MDA/2013/072)*. 2013 27.Sep.2013 [cited 2013 27.Sep]; Available from: <http://www.mhra.gov.uk/Publications/Safetywarnings/MedicalDeviceAlerts/CON316337>.

7. Department of Health Hong Kong. *Recall of cardiac implants*. 2006 28.Mar.2013 [cited 2006 27.Jun]; Available from: <http://www.mdco.gov.hk/tc_chi/safety/recalls/press_20060627.html>.

8. Department of Health Hong Kong. *Cardiac Implants Battery Issues*. 2009 28.Mar.2013 [cited 2009 08.Sep]; Available from: <http://www.mdco.gov.hk/tc_chi/safety/recalls/press_20090909.html>.

9. Swiss Agency for Therapeutic Products. *URGENT FIELD SAFETY NOTICE IMPORTANT MEDICAL DEVICE INFORMATION Concerto® CRT-D Virtuoso® ICD* 2009 [cited 2009 21.Sep]; Available from: https://[www.swissmedic.ch/recalllists_dl/02561/Vk_20090916_12-e1.pdf](http://www.swissmedic.ch/recalllists_dl/02561/Vk_20090916_12-e1.pdf).

10. Swiss Agency for Therapeutic Products. *Important Medical Device Information*. 2013 [cited 2013 09.Sep]; Available from: https://[www.swissmedic.ch/recalllists_dl/08334/Vk_20130904_05_e1.pdf](http://www.swissmedic.ch/recalllists_dl/08334/Vk_20130904_05_e1.pdf).

11. Government of Canada. *Contak Renewal TR2 CRT-P*. 2006 15.Jun.2012 [cited 2006 26.Jun]; Available from: <http://www.healthycanadians.gc.ca/recall-alert-rappel-avis/hc-sc/2006/10925r-eng.php>.

12. Swiss Agency for Therapeutic Products. *Guidant Contak Renewal 3RF and 4 RF System*. 2006 [cited 2006 20.Mar]; Available from: https://[www.swissmedic.ch/recalllists_dl/00968/Vk_20060323_03-d1.pdf](http://www.swissmedic.ch/recalllists_dl/00968/Vk_20060323_03-d1.pdf).

13. Government of Canada. *A) Alto VR625 Cardioverter Defibrillator B) Alto DR614 Cardioverter Defibrillator C) Alto 2 DR624 Cardioverter Defibrillator*. 2005 15.Jun.2012; Available from: <http://www.healthycanadians.gc.ca/recall-alert-rappel-avis/hc-sc/2005/10502r-eng.php>.

14. U.S. Food and Drug Administration. *FDA Preliminary Public Health Notification: Guidant VENTAK PRIZM® 2 DR and CONTAK RENEWAL® Implantable Cardioverter Defibrillators*. 2005 21.Mar.2013 [cited 2005 01.Jul]; Available from: <http://www.fda.gov/MedicalDevices/Safety/AlertsandNotices/PublicHealthNotifications/ucm062117.htm>.

15. Department of Health Hong Kong. *Safety issues of Medico ICDs*. 2011 28.Mar.2013 [cited 2011 27.Jan]; Available from: <http://www.mdco.gov.hk/tc_chi/safety/recalls/press_20110127.html>.

16. Swiss Agency for Therapeutic Products. *INCOR® Aktuelle Anwenderinformation AI-11-01*. 2011 [cited 2011 04.Apr]; Available from: https://[www.swissmedic.ch/recalllists_dl/04421/Vk_20110330_03-d1.pdf](http://www.swissmedic.ch/recalllists_dl/04421/Vk_20110330_03-d1.pdf).

17. UK. Medicines and Healthcare Products Regulatory Agency (MHRA). *Medtronic implantable defibrillators, models: Marquis VR 7230, Marquis DR 7274, InSync Marquis 7277*. 2004 07.Feb.2008 [cited 2004 26.Apr]; Available from: <http://www.mhra.gov.uk/Publications/Safetywarnings/MedicalDeviceAlerts/CON008561>.

18. Swiss Agency for Therapeutic Products. *Physio-Control Corporation Lifepak 20 Model Battery* [cited 2007 17.Jul]; Available from: https://[www.swissmedic.ch/recalllists_dl/01734/Vk_20070711_05-d1.pdf](http://www.swissmedic.ch/recalllists_dl/01734/Vk_20070711_05-d1.pdf).

19. Swiss Agency for Therapeutic Products. *Anwenderinformation LIFEPAK CR® Plus Defibrillator / LIFEPAK EXPRESS® Defibrillator* 2007 [cited 2007 18.Sep]; Available from: https://[www.swissmedic.ch/recalllists_dl/01827/Vk_20070828_08-d1.pdf](http://www.swissmedic.ch/recalllists_dl/01827/Vk_20070828_08-d1.pdf).

20. Swiss Agency for Therapeutic Products. *Dringende Sicherheitsinformation zum Medizinprodukt Programmiergerätesoftware Version 2.24 für Reply / Esprit Herzschrittmacher* 2011 [cited 2011 09.May]; Available from: https://[www.swissmedic.ch/recalllists_dl/04535/Vk_20110503_04-d1.pdf](http://www.swissmedic.ch/recalllists_dl/04535/Vk_20110503_04-d1.pdf).

21. Swiss Agency for Therapeutic Products. *St. Jude Medical Accent DR und Anthem CRT-P Herzschrittmacher Potentiell fehlerhafte Messwerte der Elektrodenimpedanz* 2011 [cited 2011 03.Oct]; Available from: https://[www.swissmedic.ch/recalllists_dl/05160/Vk_20110928_06_d1.pdf](http://www.swissmedic.ch/recalllists_dl/05160/Vk_20110928_06_d1.pdf).

22. Australia Therapeutic Goods Administration. *EnTrust implantable cardiac defibrillators - battery problems*. 2012 25.Jun.2012 [cited 2012 13.Apr]; Available from: <http://tga.gov.au/safety/alerts-device-entrust-icd-120413.htm#.U47jx-JpMoN>.

23. UK. Medicines and Healthcare Products Regulatory Agency (MHRA). *Subcutaneous implantable cardioverter defibrillator (S-ICD): SQ-RX® pulse generator, model 1010*. 2011 [cited 2011 14.Jun]; Available from: <http://www.mhra.gov.uk/home/groups/dts-bs/documents/medicaldevicealert/con120317.pdf>.

24. Swiss Agency for Therapeutic Products. *Guidant Dringende Medizinprodukte Sicherheitsinformation & Korrekturmaßnahmen*. 2006 [cited 2006 04.July]; Available from: https://[www.swissmedic.ch/recalllists_dl/01111/Vk_20060628_01-d1.pdf](http://www.swissmedic.ch/recalllists_dl/01111/Vk_20060628_01-d1.pdf).

25. UK. Medicines and Healthcare Products Regulatory Agency (MHRA). *Boston Scientific Ltd: Vitality and Assure implantable cardioverter defibrillator (ICD) families and Contak Renewal cardiac resynchronisation therapy defibrillator (CRT-D) families*. 2007 07.Feb.2008 [cited 2007 15.Mar]; Available from: <http://www.mhra.gov.uk/Publications/Safetywarnings/MedicalDeviceAlerts/CON2030556>.

26. UK. Medicines and Healthcare Products Regulatory Agency (MHRA). *Medical Device Alert: Implantable pacemakers manufactured by Sorin* 2013 20.Dec.2013 [cited 2013 19.Dec]; Available from: <http://www.mhra.gov.uk/home/groups/dts-bs/documents/medicaldevicealert/con355518.pdf>.

27. Swiss Agency for Therapeutic Products. *Disabled Patient Alerts in En Trust ICD during Lead Integrity Alert Software Installation and Removal*. 2008 [cited 2008 04.Apr]; Available from: https://[www.swissmedic.ch/recalllists_dl/01216/Vk_20081126_02-e1.pdf](http://www.swissmedic.ch/recalllists_dl/01216/Vk_20081126_02-e1.pdf).

28. Swiss Agency for Therapeutic Products. *St. Jude Medical Convert+ Model V-195 Implantable Cardioverter-Defibrillator* 2010 [cited 2006 17.May]; Available from: https://[www.swissmedic.ch/recalllists_dl/03261/Vk_20100512_08-e1.pdf](http://www.swissmedic.ch/recalllists_dl/03261/Vk_20100512_08-e1.pdf).

29. Swiss Agency for Therapeutic Products. *Dringende Medizinprodukte Sicherheitsinformation zu PARADYM DR, PARADYM CRT-D und PARADYM CRT-D sonR ICD* 2010 [cited 2010 28.Jun]; Available from: https://[www.swissmedic.ch/rueckrufe_medizinprodukte/archiv/index.html?lang=en&RlArchiv=2010-01#top](http://www.swissmedic.ch/rueckrufe_medizinprodukte/archiv/index.html?lang=en&RlArchiv=2010-01#top).

30. Swiss Agency for Therapeutic Products. *MEDICAL DEVICE CORRECTION Consulta® CRT-D, Secura® DR/VR, Concerto® II CRT-D, Virtuoso® II DR/VR, Maximo® II CRT-D, Maximo® II DR/VR* 2010 [cited 2010 25.May]; Available from: https://[www.swissmedic.ch/recalllists_dl/03298/Vk_20100512_02-e1.pdf](http://www.swissmedic.ch/recalllists_dl/03298/Vk_20100512_02-e1.pdf).

31. Swiss Agency for Therapeutic Products. *Halbautomatischer externer Defibrillator LIFEPAK® 500 Modell 97401, 98401 und 99401* 2007 [cited 2007 10.Jul]; Available from: https://[www.swissmedic.ch/recalllists_dl/01729/Vk_20070627_02-d1.pdf](http://www.swissmedic.ch/recalllists_dl/01729/Vk_20070627_02-d1.pdf).

32. Swiss Agency for Therapeutic Products. *Wichtige Information für Ärzte Vitatron Herzschrittmacher der C- und T-Serie* 2007 [cited 2007 06.Mar]; Available from: https://[www.swissmedic.ch/recalllists_dl/01490/Vk_20070213_05-d1.pdf](http://www.swissmedic.ch/recalllists_dl/01490/Vk_20070213_05-d1.pdf).

33. Department of Health Hong Kong. *Software issues of Sorin ICDs*. 2010 28.Mar.2013 [cited 2010 15.Jun]; Available from: <http://www.mdco.gov.hk/tc_chi/safety/recalls/press_20100615.html>.

34. Australia Therapeutic Goods Administration. *St Jude Medical Implantable Cardiac Devices when used with Merlin programmer and version 17.2.2 software*. 2014 [cited 2014 28.Jan]; Available from: <http://www.tga.gov.au/safety/alerts-device-st-jude-medical-icd-crtd-140128.htm#.U5BYzuJpMoM>.

35. China Food and Drug Administration. *Recall of ICD*. 2011 [cited 2011 16.May]; Available from: <http://www.sfda.gov.cn/WS01/CL0861/61613.html>.

36. Swiss Agency for Therapeutic Products. *St. Jude Medical Accent SR Model PM1110 and Accent DR Model 2112 Pacemakers Potential for the Inability to Provide Rate Responsive Sensor Driven Pacing Rates*. 2012 [cited 2012 07.Dec]; Available from: https://[www.swissmedic.ch/recalllists_dl/07073/Vk_20121212_02-e1.pdf](http://www.swissmedic.ch/recalllists_dl/07073/Vk_20121212_02-e1.pdf).

37. Swiss Agency for Therapeutic Products. *Wichtige Sicherheitsinformation und Abhilfemassnahme für Medizinisches Gerät AEDs der Defibtech-LIFELINE-Serie*. 2007 [cited 2007 08.May]; Available from: https://[www.swissmedic.ch/recalllists_dl/01639/Vk_20070315_02-d1.pdf](http://www.swissmedic.ch/recalllists_dl/01639/Vk_20070315_02-d1.pdf).

38. Department of Health Hong Kong. *Software issues of ICDs*. 2006 28.Mar.2013 [cited 2006 19.Oct]; Available from: <http://www.mdco.gov.hk/tc_chi/safety/recalls/press_20061019.html>.

39. UK. Medicines and Healthcare Products Regulatory Agency (MHRA). *Medical Device Alert: Implantable pacemakers manufactured by Medtronic (MDA/2011/097)*. 2011 [cited 2011 28.Sep]; Available from: <http://www.mhra.gov.uk/home/groups/dts-bi/documents/medicaldevicealert/con129226.pdf>.

40. Swiss Agency for Therapeutic Products. *APS III Programmer Merlin PCS Programmer Identity Pacemaker* 2006 [cited 2006 07.Nov]; Available from: https://[www.swissmedic.ch/recalllists_dl/01316/Vk_20061019_04-e1.pdf](http://www.swissmedic.ch/recalllists_dl/01316/Vk_20061019_04-e1.pdf).

41. Australia Therapeutic Goods Administration. *Medtronic CareLink 2090 Programmer (Implantable pacemaker programmer)*. 2013 [cited 2013 03.Oct]; Available from: <http://www.tga.gov.au/SARA/arn-detail.aspx?k=RC-2013-RN-01024-1>.

42. Government of Canada. *En Trust ICD*. 2005 15.Jun.2012 [cited 2005 17.May]; Available from: <http://www.healthycanadians.gc.ca/recall-alert-rappel-avis/hc-sc/2005/10656r-eng.php>.

43. Swiss Agency for Therapeutic Products. *Safety Information Technical Bulletin*. 2008 [cited 2008 10.Mar]; Available from: https://[www.swissmedic.ch/recalllists_dl/00437/Vk_20080226_09-e1.pdf](http://www.swissmedic.ch/recalllists_dl/00437/Vk_20080226_09-e1.pdf).

44. Swiss Agency for Therapeutic Products. *IMPORTANT MEDICAL DEVICE SAFETY INFORMATION AND CORRECTIVE ACTIONS REGARDING CERTAIN ELA MEDICAL SYMPHONY AND RHAPSODY PACEMAKERS*. 2005 [cited 2005 09.Nov]; Available from: https://[www.swissmedic.ch/recalllists_dl/00076/Vk_20051026_06-e1.pdf](http://www.swissmedic.ch/recalllists_dl/00076/Vk_20051026_06-e1.pdf).

45. Department of Health Hong Kong. *Field Safety Notice: HeartWare™ Ventricular Assist System*. 2012 15.Dec.2012 [cited 2012 04.Jan]; Available from: <http://www.mdco.gov.hk/english/safety/recalls/recalls_20120104b.html>.

46. Swiss Agency for Therapeutic Products. *HeartWare Urgent Field Safety Notice*. 2013 [cited 2013 24.Jun]; Available from: https://[www.swissmedic.ch/recalllists_dl/07914/Vk_20130611_03-e1.pdf](http://www.swissmedic.ch/recalllists_dl/07914/Vk_20130611_03-e1.pdf).

47. Swiss Agency for Therapeutic Products. *Field Safety Notice HeartWare® Ventricular Assist System* 2011 [cited 2011 27.Dec]; Available from: https://[www.swissmedic.ch/recalllists_dl/05534/Vk_20111221_07-e1.pdf](http://www.swissmedic.ch/recalllists_dl/05534/Vk_20111221_07-e1.pdf).

48. Government of Canada. *Important Safety information on certain St. Jude Medical implantable cardiac defibrillators*. 2005 08.Feb.2013 [cited 2005 06.Oct]; Available from: <http://www.healthycanadians.gc.ca/recall-alert-rappel-avis/hc-sc/2005/14362a-eng.php>.

49. UK. Medicines and Healthcare Products Regulatory Agency (MHRA). *MDA/2005/045 - St Jude Medical implantable cardioverter defibrillators (ICDs)*. 2005 07.Jan.2008 [cited 2005 25.Jul]; Available from: <http://www.mhra.gov.uk/PrintPreview/PublicationSP/CON1004103>.

50. UK. Medicines and Healthcare Products Regulatory Agency (MHRA). *Medical Device Alert: Isoline implantable cardioverter defibrillator (ICD) leads manufactured by Sorin Group Italia Srl (MDA/2013/007)*. 2013 [cited 2013 22.Feb]; Available from: <http://www.mhra.gov.uk/home/groups/dts-bs/documents/medicaldevicealert/con239427.pdf>.

51. Swiss Agency for Therapeutic Products. *Product Advisory-This Advisory only applies to CONTAK RENEWAL 4 RF/4 RF HE CRT-D Devices Programmed to LV-only Pacing*. 2005 [cited 2007 01.May]; Available from: https://[www.swissmedic.ch/recalllists_dl/00640/Vk_20080508_01-d1.pdf](http://www.swissmedic.ch/recalllists_dl/00640/Vk_20080508_01-d1.pdf).

52. Swiss Agency for Therapeutic Products. *Medical Device Alert: Implantable cardioverter defibrillator (ICD) manufactured by Sorin CRM (MDA/2011/073)*. 2011 05.Jul.2011 [cited 2011 29.Jun]; Available from: <http://www.mhra.gov.uk/PrintPreview/MedicalDeviceAlertSP/CON120446?tabName=allTabs>.

53. Swiss Agency for Therapeutic Products. *Biphasic LIFEPAK® 12 Defibrillator/Monitor series Medical Device Correction* 2008 [cited 2008 02.Jun]; Available from: https://[www.swissmedic.ch/recalllists_dl/00541/Vk_20080530_02-e1.pdf](http://www.swissmedic.ch/recalllists_dl/00541/Vk_20080530_02-e1.pdf).

54. Swiss Agency for Therapeutic Products. *Anwenderinformation Dringender Sicherheitshinweis LIFEPAK® 12 und LIFEPAK 20 Defibrillator/Monitor* 2008 [cited 2008 07.Jul]; Available from: https://[www.swissmedic.ch/recalllists_dl/00640/Vk_20080508_01-d1.pdf](http://www.swissmedic.ch/recalllists_dl/00640/Vk_20080508_01-d1.pdf).

55. Swiss Agency for Therapeutic Products. *Product Advisory-Inappropriate therapy associated with certain right ventricular (RV) lead complications may occur more frequently if the Respiratory Sensor is programmed on*. 2009 [cited 2009 31.Mar]; Available from: https://[www.swissmedic.ch/recalllists_dl/02124/Vk_20090324_03-e1.pdf](http://www.swissmedic.ch/recalllists_dl/02124/Vk_20090324_03-e1.pdf).

56. Department of Health Hong Kong. *Field Safety Notice: Impulse Dynamics OPTIMIZER® III Implantable Pulse Generator*. 2011 15.Dec.2012 [cited 2011 25.Nov]; Available from: <http://www.mdco.gov.hk/english/safety/recalls/recalls_20111125.html>.

57. Swiss Agency for Therapeutic Products. *Wichtiger Hinweis zu einer selten angewendeten subpektoralen Implantationstechnik von VITALITY®HE implantierbare Cardioverter Defibrillatoren (ICDs) und CONTAK RENEWAL® 3 und 4 Defibrillatoren zur Herzresynchronisationstherapie (CRT-Ds).* . 2006 [cited 2006 31.May]; Available from: https://[www.swissmedic.ch/recalllists_dl/01067/Vk_20060518_01-d1.pdf](http://www.swissmedic.ch/recalllists_dl/01067/Vk_20060518_01-d1.pdf).

58. Swiss Agency for Therapeutic Products. *Aktualisierung des Sicherheitshinweises vom Mai 2006 zu möglichen Störungen bei subpektor aler Implantation mit Seriennummern nach unten zeigend bei CONTAK RENEWAL 4 und VITALITY Systemen* 2008 [cited 2008 28.Jan]; Available from: https://[www.swissmedic.ch/recalllists_dl/00389/Vk_20080109_05-d1.pdf](http://www.swissmedic.ch/recalllists_dl/00389/Vk_20080109_05-d1.pdf).

59. UK. Medicines and Healthcare Products Regulatory Agency (MHRA). *Guidant implantable cardioverter defibrillators (ICDs) and cardiac resynchronization therapy defibrillators (CRT-Ds)*. 2005 07.Feb.2008 [cited 2005 30.Jun]; Available from: <http://www.mhra.gov.uk/Publications/Safetywarnings/MedicalDeviceAlerts/CON1004088>.

60. Swiss Agency for Therapeutic Products. *Urgent Medical Device Recall Carotid Wallstent Monorail carotid endoprosthesis*. 2006 [cited 2006 19.Jul]; Available from: https://[www.swissmedic.ch/recalllists_dl/01148/Vk_20060712_07-e1.pdf](http://www.swissmedic.ch/recalllists_dl/01148/Vk_20060712_07-e1.pdf).

61. U.S. Food and Drug Administration. *Medtronic Inc. Sprint Fidelis® Defibrillator Leads*. 2007 02.May.2014 [cited 2007 15.Oct]; Available from: <http://www.fda.gov/MedicalDevices/Safety/ListofRecalls/ucm062377.htm>.

62. Government of Canada. *A) Bard Luminexx 3 Vascular Stent B) Bard Luminexx 3 Vascular Stent* 2005 15.Jun.2012 [cited 2005 25.Jul]; Available from: <http://www.healthycanadians.gc.ca/recall-alert-rappel-avis/hc-sc/2005/10641r-eng.php>.

63. Swiss Agency for Therapeutic Products. *Urgent Field Safety Notice Product Recall Bard Lifestent Vascular Stent System*. 2011 [cited 2011 17.Jan]; Available from: https://[www.swissmedic.ch/recalllists_dl/04120/Vk_20110111_10-e1.pdf](http://www.swissmedic.ch/recalllists_dl/04120/Vk_20110111_10-e1.pdf).

64. Swiss Agency for Therapeutic Products. *Urgent Field Safety Notice Voluntary recall concerning E-vita THORACIC 3G Stentgraft System*. 2013 [cited 2013 25.Nov]; Available from: https://[www.swissmedic.ch/recalllists_dl/08770/Vk_20131120_25_e1.pdf](http://www.swissmedic.ch/recalllists_dl/08770/Vk_20131120_25_e1.pdf).

65. Swiss Agency for Therapeutic Products. *Field Safety Notice Urgent Medical Device Recall NexStent Carotid Stent & Monorail Delivery System*. 2008 [cited 2008 23.Jun]; Available from: https://[www.swissmedic.ch/recalllists_dl/00603/Vk_20080617_04-e1.pdf](http://www.swissmedic.ch/recalllists_dl/00603/Vk_20080617_04-e1.pdf).

66. Swiss Agency for Therapeutic Products. *Urgent Field Safety Notice Device Model: Axxess Drug Eluting Coronary Bifurcation Stent System*. 2013 [cited 2013 11.Dec]; Available from: https://[www.swissmedic.ch/recalllists_dl/08945/Vk_20131213_06-e1.pdf](http://www.swissmedic.ch/recalllists_dl/08945/Vk_20131213_06-e1.pdf).

67. Swiss Agency for Therapeutic Products. *Urgent Medical Device Recall NexStent Monorail Carotid Stent System*. 2006 [cited 2006 07.Jun]; Available from: https://[www.swissmedic.ch/recalllists_dl/01074/Vk_20060530_03-e1.pdf](http://www.swissmedic.ch/recalllists_dl/01074/Vk_20060530_03-e1.pdf).

68. Swiss Agency for Therapeutic Products. *Important Customer Notification NexStent Carotid Stent and Monorail Delivery System*. 2007 [cited 2007 08.May]; Available from: https://[www.swissmedic.ch/recalllists_dl/01647/Vk_20070508_02-e1.pdf](http://www.swissmedic.ch/recalllists_dl/01647/Vk_20070508_02-e1.pdf).

69. U.S. Food and Drug Administration. *Boston Scientific Taxus™ Express2TM Coronary Stent*. 2004 02.May.2014 [cited 2004 01.Jul]; Available from: <http://www.fda.gov/MedicalDevices/Safety/ListofRecalls/ucm064778.htm>.

70. U.S. Food and Drug Administration. *Boston Scientific Express2TM (bare metal) Coronary Stent*. 2004 02.May.2014 [cited 2004 16.Jul]; Available from: <http://www.fda.gov/MedicalDevices/Safety/ListofRecalls/ucm064772.htm>.

71. U.S. Food and Drug Administration. *Bard LifeStent Solo Vascular Stent - Failure to Deploy Stent*. 2013 18.Oct.2013 [cited 2013 30.Sep]; Available from: <http://www.fda.gov/MedicalDevices/Safety/ListofRecalls/ucm371318.htm>.

72. Department of Health Hong Kong. *FDA Class I Recall: Boston Scientific Innova™ Self-Expanding Stent System*. 2011 15.Dec.2012 [cited 2011 02.Sep]; Available from: <http://www.mdco.gov.hk/english/safety/recalls/recalls_20110902.html>.

73. Swiss Agency for Therapeutic Products. *Notification for a recall regarding certain references of Coronary Stents Tsunami Gold*. 2006 [cited 2006 11.Jan]; Available from: https://[www.swissmedic.ch/recalllists_dl/00276/Vk_20060105_11-e1.pdf](http://www.swissmedic.ch/recalllists_dl/00276/Vk_20060105_11-e1.pdf).

74. U.S. Food and Drug Administration. *Guidant Corp. Pacemakers*. 2005 02.Jun.2014 [cited 2005 18.Jul]; Available from: <http://www.fda.gov/MedicalDevices/Safety/ListofRecalls/ucm063722.htm>.

75. Irish Medicines Board. *IntraStent Unmounted Balloon Expandable Stent*. 2008 [cited 2008 24.Jan]; Available from: <http://www.imb.ie/EN/Safety--Quality/Advisory-Warning--Recall-Notices/Medical-Devices/IntraStent-Unmounted-Balloon-Expandable-Stent.aspx?page=1&noticetypeid=-1&year=2008>.

76. Swiss Agency for Therapeutic Products. *Urgent Field Safety Notice of Potential Stent Expansion Uniformity Out of Specification Condition*. 2011 [cited 2011 20.Jue]; Available from: https://[www.swissmedic.ch/rueckrufe_medizinprodukte/archiv/index.html?lang=en&RlArchiv=2011-01](http://www.swissmedic.ch/rueckrufe_medizinprodukte/archiv/index.html?lang=en&RlArchiv=2011-01).

77. Australia Therapeutic Goods Administration. *Medtronic Mosaic Porcine Aortic Bioprosthesis*. 2014 [cited 2014 28.Jan]; Available from: <http://www.tga.gov.au/safety/alerts-device-medtronic-bioprosthesis-140128.htm#.U5mNduJpMoM>.

78. SWiss Agency for Therapeutic Products. *Mechanical stress associated with subpectoral implantation may weaken the bond between the header and the titanium case in COGNIS® cardiac resynchronization therapy defibrillators (CRT-Ds) and TELIGEN® implantable cardioverter defibrillators (ICDs)*. 2009 [cited 2009 07.Dec]; Available from: https://[www.swissmedic.ch/recalllists_dl/02784/Vk_20091203_06-e1.pdf](http://www.swissmedic.ch/recalllists_dl/02784/Vk_20091203_06-e1.pdf).

79. Swiss Agency for Therapeutic Products. *Medtronic CoreValve® Delivery Catheter System (DCS) Recall*. 2011 [cited 2011 15.Aug]; Available from: https://[www.swissmedic.ch/recalllists_dl/04741/Vk_20110803_05-e1.pdf](http://www.swissmedic.ch/recalllists_dl/04741/Vk_20110803_05-e1.pdf).

80. Swiss Agency for Therapeutic Products. *Urgent Medical Device Correction HeartWare® Ventricular Assist System*. 2013 [cited 2013 23.Dec]; Available from: https://[www.swissmedic.ch/recalllists_dl/08932/Vk_20131217_01_e1.pdf](http://www.swissmedic.ch/recalllists_dl/08932/Vk_20131217_01_e1.pdf).

81. U.S. Food and Drug Administration. *Medtronic Inc., Kappa 600/700/900 Series of Pacemakers and Sigma 100/200/300 Series of Pacemakers*. 2009 02.Jun.2014 [cited 2009 18.May]; Available from: <http://www.fda.gov/MedicalDevices/Safety/ListofRecalls/ucm166344.htm>.

82. Government of Canada. *Important Safety Information on Medtronic Sigma Series pacemakers- Medtronic of Canada Ltd. - For Health Professionals*. 2005 06.Feb.2013 [cited 2005 30.Nov]; Available from: <http://healthycanadians.gc.ca/recall-alert-rappel-avis/hc-sc/2005/14355a-eng.php>.

83. Department of Health Hong Kong. *Recall of Medtronic International Ltd ICD*. 2009 28.Mar.2013 [cited 2009 19.May]; Available from: <http://www.mdco.gov.hk/tc_chi/safety/recalls/press_20090519.html>.

84. Department of Health Hong Kong. *Recall of Medtronic Sigma® ICD*. 2005 28.Mar.2013 [cited 2005 01.Dec]; Available from: <http://www.mdco.gov.hk/tc_chi/safety/recalls/press_20051201.html>.

85. Department of Health Hong Kong. *Field Safety Notice: Thoratec HeartMate II® Left Ventricular Assist System*. 2012 [cited 2012 28.Dec.2012]; Available from: <http://www.mdco.gov.hk/english/safety/recalls/recalls_20120228a.html>.

86. U.S. Food and Drug Administration. *St. Jude Medical, Riata and Riata ST Silicone Endocardial Defibrillation Leads*. 2011 02.Jun.2014 [cited 2011 28.Nov]; Available from: <http://www.fda.gov/MedicalDevices/Safety/ListofRecalls/ucm284360.htm>.

87. Swiss Agency for Therapeutic Products. *Urgent Medical Device Field Safety Notice ISOLINE defibrillation leads, model 2CR5, 2CR6 and 2CT6* 2013 [cited 2013 28.Jan]; Available from: https://[www.swissmedic.ch/recalllists_dl/07246/Vk_20130130_10-e1.pdf](http://www.swissmedic.ch/recalllists_dl/07246/Vk_20130130_10-e1.pdf).

88. UK. Medicines and Healthcare Products Regulatory Agency (MHRA). *Medical Device Alert: Implantable cardioverter defibrillator (ICD) leads Manufactured by St Jude Medical (MDA/2012/061)* 2012 [cited 2012 10.Sep]; Available from: <http://www.mhra.gov.uk/Publications/Safetywarnings/MedicalDeviceAlerts/CON184434>.

89. UK. Medicines and Healthcare Products Regulatory Agency (MHRA). *Medical Device Alert: Left ventricular cardiac resynchronization therapy (CRT) leads manufactured by St Jude Medical (MDA/2012/021)*. 2012 [cited 2012 24.Apr]; Available from: <http://www.mhra.gov.uk/Publications/Safetywarnings/MedicalDeviceAlerts/CON149817>.

90. Swiss Agency for Therapeutic Products. *IMPORTANT PRODUCT INFORMATION St. Jude Medical Riata and Riata ST Silicone Endocardial Leads*. 2010 [cited 2010 27.Dec]; Available from: https://[www.swissmedic.ch/recalllists_dl/04059/Vk_20101223_10_e1.pdf](http://www.swissmedic.ch/recalllists_dl/04059/Vk_20101223_10_e1.pdf).

91. U.S. Food and Drug Administration. *Medtronic Interventional Guidewires and ATTAIN HYBRID® Guidewires*. 2013 20.Nov.2013 [cited 2013 21.Oct]; Available from: <http://www.fda.gov/MedicalDevices/Safety/ListofRecalls/ucm375659.htm>.

92. Swiss Agency for Therapeutic Products. *Field Safety Notice Urgent Medical Device Recall Back-up Meier Steerable Guidewire*. 2010 [cited 2010 03.May]; Available from: https://[www.swissmedic.ch/recalllists_dl/03205/Vk_20100427_03-e1.pdf](http://www.swissmedic.ch/recalllists_dl/03205/Vk_20100427_03-e1.pdf).
